# Value of dynamic contrast enhanced MRI in differential diagnostics of Warthin tumors and parotid malignancies

**DOI:** 10.1038/s41598-021-95820-y

**Published:** 2021-08-11

**Authors:** Bogusław Mikaszewski, Karolina Markiet, Aneta Smugała, Dominik Stodulski, Ewa Garsta, Jakub Piątkowski, Edyta Szurowska

**Affiliations:** 1 Department of Otolaryngology, Faculty of Medicine, Medical University of Gdansk, 17 Smoluchowskiego Street, 80-214 Gdańsk, Poland; 2 2nd Department of Radiology, Faculty of Health Sciences, Medical University of Gdansk, 17 Smoluchowskiego Street, 80-214 Gdańsk, Poland; 3 Department of Radiology, University Clinical Center, Gdansk, 17 Smoluchowskiego Street, 80-214 Gdańsk, Poland

**Keywords:** Salivary gland diseases, Cancer imaging, Head and neck cancer

## Abstract

To define an algorithm for differential diagnostics of parotid malignancies and Warthin tumors (WTs) based on dynamic contrast enhanced MRI (DCE-MRI). 55 patients with parotid tumors treated surgically were analyzed. Of which, 19 had parotid malignancy and 36 had WTs confirmed with postoperative histopathological examination. Accuracy of DCE-MRI parameters (T_peak_ and WR) was compared with the histopathological diagnosis. ROC analysis was performed to determine sensitivity and specificity of DCE-MRI with various T_peak_ and WR cut-off values. WT showed significantly lower median T_peak_ and higher median WR than malignant lesions. The cut-off values for T_peak_ and WR providing maximum sensitivity (84.2%) and specificity (86.1%) for malignant tumors were T_peak_ > 60 s and WR ≤ 30%. Different diagnostic algorithm, i.e., lower cut-off value for T_peak_ (T_peak_ = 60 s), increases sensitivity of DCE-MRI in differentiating parotid malignancies from WTs. However, WR > 30% seems to be a key diagnostic criterion for benign lesions. Precise and reliable preoperative diagnostics of parotid tumors aids in careful surgical planning, thereby assisting in achieving sufficient surgical resection margins and facial nerve preservation.

## Introduction

Parotid tumors represent approximately 5% of all head and neck neoplasms^1,2^. Warthin tumors (WTs), also referred to as papillary cystadenoma lymphomatosum, is the second most common benign tumor of the parotid found in 14–30% of the patients. It usually occurs in the 6th–7th decade of life with a greater incidence in smokers than non-smokers^3–6^. As benign slowly growing lesions that rarely recur or undergo malignant transformation, WTs are often treated less radically with local excision or even expectant management^6–9^. However, such therapeutic decisions can only be made when malignancy is unequivocally excluded.

Fine needle biopsy remains a standard in preoperative evaluation of parotid tumors^10^. However, this method has limited application in the case of small and/or deeply located lesions^11–13^, and if performed incorrectly may result in systemic spread of cancer cells^14^ as well as other complications^3^. Moreover, fine needle biopsy has been shown to be less accurate in distinguishing WTs from parotid malignancies^7^. In an era of minimizing the invasiveness of surgical treatment, alternative methods are being sought to accurately differentiate between benign and malignant lesions, while also reducing patient suffering and therapeutic costs. Since previous attempts to utilize conventional MRI to distinguish WTs from parotid malignancies have proven unsuccessful^15^, great hopes are being pinned on the application of novel dynamic imaging techniques. The results from previous research imply that dynamic contrast enhanced MRI (DCE-MRI) can be applied to differentiate parotid tumors. This is primarily based on the finding that time-signal intensity curves (TICs) for malignant tumors and the two commonest benign lesions, pleomorphic adenomas (PAs) and WTs, differ markedly, especially in terms of the time to peak enhancement (T_peak_) and washout rate values (WR). According to literature, PAs show a gradual enhancement (high T_peak_) followed by a slow contrast washout (low WR)^14,16–18^. In contrast, WTs and malignant lesions are characterized by an early enhancement (low T_peak_) followed by a rapid^17,19,20^ or gradual washout, respectively^21,22^, which results in high or low WR. However, majority of these studies were limited due to inclusion of relatively small cohorts, providing inconclusive results, especially with regards to T_peak_ cut-off values for achieving optimal accuracy in distinguishing WTs from parotid malignancies.

We verified the accuracy of the existing radiological criteria for differentiating between WTs and parotid malignancies, using our relatively large database of patients subjected to preoperative DCE-MRI and treated surgically for parotid tumors. The aim of this study was to define an optimal algorithm for the differential diagnostics of these two groups of lesions on the basis of DCE-MRI.

## Material and methods

This study included 100 consecutive patients with parotid tumors, treated surgically at the Department of Otolaryngology, Medical University of Gdansk, between 2013 and 2014. 41 men and 59 women aged between 18 and 88 years (mean age 56.1 ± 15.8 years) were included in the analysis. The protocol of the study was approved by the Local Bioethics Committee at the Medical University of Gdansk, and all patients gave their written informed consent to participate in the project. Authors confirm that all research was performed in accordance with relevant guidelines/regulations. Research was performed in accordance with the Declaration of Helsinki.

Prior to the surgery, all patients underwent fine needle biopsy of the parotid tumor. Both biopsy and surgical specimens were subjected to routine cytological and histological examination at the Department of Pathomorphology, Medical University of Gdansk. Moreover, all patients routinely underwent preoperative multiparametric MRI.

### Image acquisition and processing

All MRI examinations were performed using a 1.5 T scanner (Magnetom Aera, Siemens, Erlangen, Germany) using a head coil. Table 1 displays the applied MRI examination protocol. To obtain contrast-enhanced sequences, gadolinium-based contrast agent, gadobutrol, (Gadovist, Bayer Schering Pharma, Berlin, Germany) was utilized at standard dose of 0.1 mmol/body weight (0.1 ml/body weight) at a rate of 2–3 ml/s, followed by a 20-ml saline flush. No adverse reactions occurred following contrast administration.

**Table 1:** MRI examination protocol.

**Pre-contrast sequences**
1. T2 Bl Sag^1^
2. T1 TSE^2^ Sag
3. T2 TIRM^3^ Cor^4^
4. T1 SE^5^ Cor
5. T2 TSE Tra^6^
6. T1 TSE Tra
7. T1 TSE FS^7^ Tra
8. DWI^8^ Cor (b 0 100 300 500 800) with generation of ADC^9^ map
**Gadolinium contrast-enhanced sequences**
9. T1 Vibe^10^ Dyn^11^ Tra
10. T1 TSE Tra CM^12^
11. T1 SE Cor CM
12. T1 TSE FS Tra CM
13. T1 TSE Sag CM

^1^Sag, sagittal; ^2^TSE, Turbo Spin Echo; ^3^TIRM, Turbo Inversion Recovery Magnitude; ^4^Cor, coronal; ^5^SE, Spin Echo; ^6^Tra, transverse; ^7^FS, Fat saturation; ^8^DWI, diffusion weighted imaging; ^9^ADC, apparent diffusion coefficient; ^10^Vibe, volumetric interpolated breath-hold examination; ^11^ dyn, dynamic imaging post intravenous contrast agent administration; ^12^CM, contrast medium.

Diffusion restriction was evaluated qualitatively based on increasing signal intensity with growing *b* factor in DWI sequence, along with low apparent diffusion coefficient (ADC) values. ADC values were measured using ADC maps, which were generated automatically with commercially available Siemens software (SyngoVia) by manually placing the region of interest (ROI) over the tumor area.

Dynamic contrast enhanced sequences were obtained with 36 repetitions over 226 s. DCE-MRI analysis was based on time-signal intensity curves (TIC) obtained with the above mentioned software (Siemens SyngoVia, MeanCurve tool) by placing ROI over the lesion. In the case of heterogeneous lesions, ROI was carefully placed to exclude cystic/necrotic/calcified areas and blood vessels. The mean size of ROI was approximately 3–4 mm. The MeanCurve tool provided graphical as well as numerical representation of the enhancement pattern, which enabled further mathematical calculations of T_peak_ and WR.

### Image analysis

All radiological images and obtained data were evaluated by two independent radiologists with prior experience in Head and Neck Radiology. The radiologists were blinded to the clinical history of the patient, and results of fine needle biopsy and histopathological analysis. The number and topography of the lesions as well as their morphology, including tumor size, signal intensity on T2-, T1-weighted and T1-weighted images with fat saturation and homogeneity were assessed. Additionally, ADC values for the tumor and normal parotid were measured and enhancement pattern assessed with calculation of TICs in order to establish a radiological diagnosis. The obtained TICs were classified according to criteria utilized by Yabuuchi et al.^23^, on the basis of T_peak_ and WR: A) gradual enhancement (T_peak_ > 120 s, WR < 10%, typical for 75% of PA and other adenomas), B) early enhancement and high washout (T_peak_ < 120 s, WR > 30%, typical for WTs), C) early enhancement and low washout (T_peak_ < 120 s, WR < 30%, characteristic for malignant tumors), and D) no enhancement (flat curve, specific for cystic lesions). Presence of lymphadenopathy and signs of perineural spread was also reported.

### Statistical analysis

Normal distribution of continuous variables was verified using the Kolmogorov–Smirnov test. Depending on the type of distribution, statistical characteristics of continuous variables were presented either as arithmetic means and standard deviations (SD) or medians and ranges. Statistical characteristics of discrete variables were presented as distributions of numbers and percentages. Significance of intergroup differences in the characteristics of continuous variables was verified using the Student’s t-test or Mann–Whitney U test, while Pearson’s chi-squared test or Fisher’s exact test were used for intergroup comparisons of discrete variables. Accuracy of DCE-MRI parameters (T_peak_ and WR) in differential diagnostics of WTs and malignant lesions was determined in relation to the gold standard, i.e., histological diagnosis. ROC analysis was conducted to determine sensitivity and specificity of DCE-MRI with various cut-off values for T_peak_ and WR, as well as the area under ROC curve (AUC) and its 95% confidence interval (95% CI). The accuracy of DCE-MRI with various combinations of cut-off values for T_peak_ and WR was determined on the basis of expected values from bivariate logistic regression analysis. All calculations were carried out using Statistica 10 package (StatSoft, USA), with the threshold of statistical significance set at *p* ≤ 0.05.

### Ethical approval

All procedures performed in this studu involving human participants were in accordance with the ethical standards of the institutional and/or national research committee and with the 1964 Helsinki declaration and its later amendments or comparable ethical standards. The protocol of the study was approved by the Local Bioethics Committee at the Medical University of Gdansk (NKBBN/591/2013).

### Informed consent

Informed consent was obtained from all individual participants included in the study.

## Results

One hundred patients with parotid tumors, operated between 2013 and 2014, were included in this study. Of these, 19 individuals had postoperative histopathological examination confirmed presence of malignant lesion (Table 2) while 36 were diagnosed with WTs. This subset of patients comprised of 31 men and 24 women aged between 25 and 88 years (mean age 62.2 ± 13.5). The lesions eventually identified as WTs were characterized by significantly lower median T_peak_ (40.25 s [range 31.90–209.58 s] *vs* 181.48 s [39.61–255.32 s], *p* < 0.001) and significantly higher median washout rate (30.17% [1.85–41.29%] *vs* 2.91% [0–35.7%], *p* < 0.001) than malignant lesions.

**Table 2:** Distribution of malignant parotid tumors identified in the analyzed material according to their microscopic type.

Histological type	n (%)
Adenocarcinoma	3 (15.8%)
Acinic cell carcinoma	2 (10.5%)
Adenoid cystic carcinoma	2 (10.5%)
Myoepithelial carcinoma	2 (10.5%)
Metastasis to lymph node	2 (10.5%)
Epithelial myoepithelial carcinoma	1 (5.3%)
Salivary duct carcinoma	1 (5.3%)
Mucoepidermoid carcinoma	1 (5.3%)
Carcinoma ex pleomorphic adenoma	1 (5.3%)
Adenoid cell carcinoma	1 (5.3%)
Metastatic clear cell carcinoma	1 (5.3%)
Recurrent carcinoma after RT	1 (5.3%)
Marginal zone B-cell lymphoma	1 (5.3%)

**Figure. 1:**
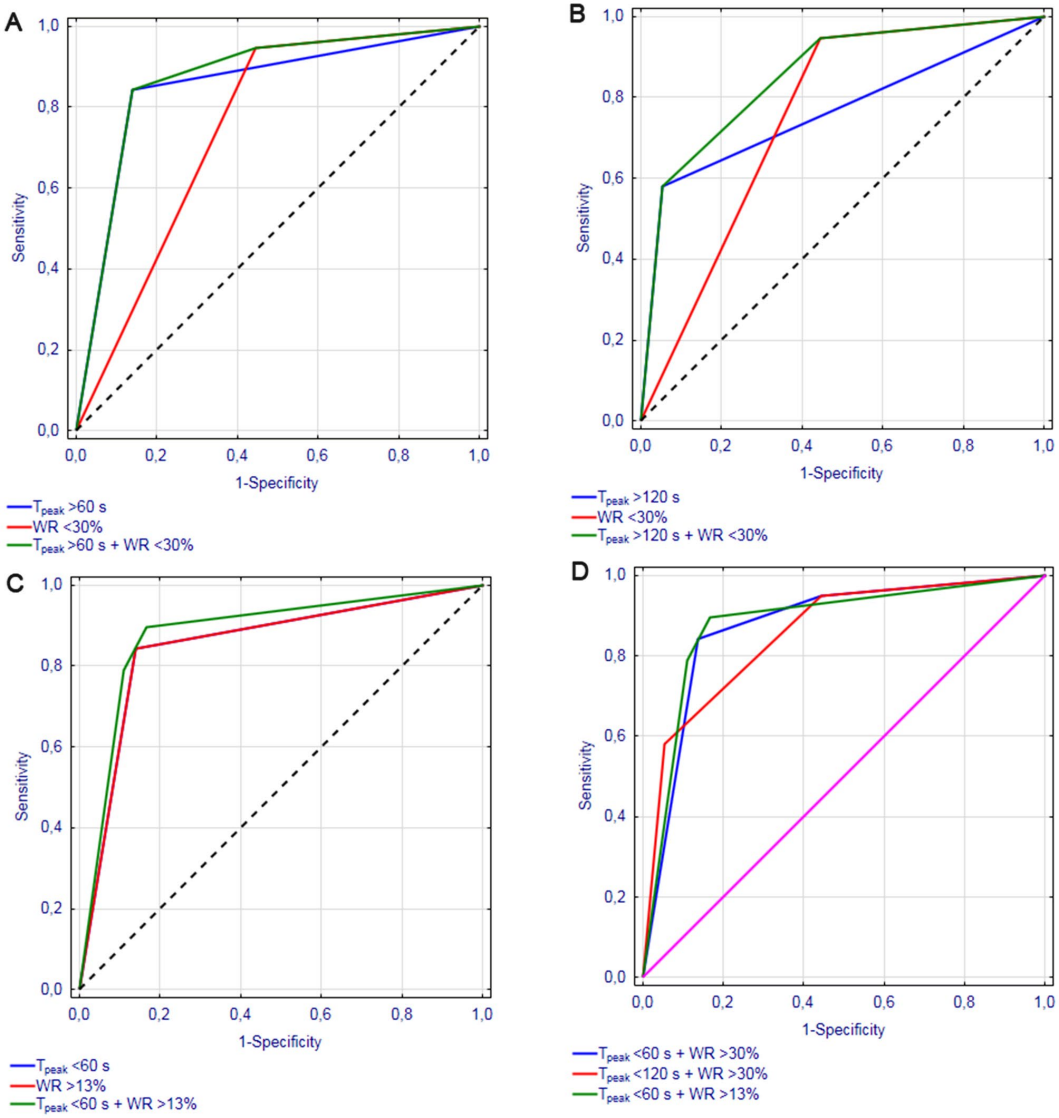
ROC curves illustrating the accuracy of DCE-MRI with the cut-off values (**A**) T_peak_ > 60 s and/or WR ≤ 30%, (**B**) T_peak_ > 120 s and/or WR ≤ 30%, (**C**) T_peak_ > 60 s and/or WR ≤ 13% in the differential diagnostics of parotid malignancies, and (**D**) comparison of the ROC curves for A-C.

During the first stage of the analysis, the accuracy of T_peak_ = 60 s and T_peak_ = 120 s for differentiating WTs from parotid malignancies was compared. These cut-off values for T_peak_ were previously used by Takashima et al.^24^ (T_peak_ = 60 s), Yabuuchi et al.^23^ and Hisatomi et al.^14^ (T_peak_ = 120 s). Using cut-off values of T_peak_ = 60 s and T_peak_ = 120 s, we correctly identified 16/19 and 11/19 malignant tumors, respectively, and 31/36 and 34/36 WTs, respectively. Upon analyzing the distributions of malignant lesions and WTs within subgroups identified based on these cut-off values, we found that both criteria were able to accurately distinguish between these histological types (*p* < 0.001) (Table 3). ROC analysis showed that DCE-MRI with cut-off value T_peak_ = 60 s provided 84.2% sensitivity and 86.1% specificity for identification of malignant tumors (AUC = 0.852, 95% CI 0.736–0.968). While the sensitivity and specificity with cut-off value T_peak_ = 120 s were 57.9% and 94.4%, respectively (AUC = 0.762, 95% CI 0.613–0.910) (Fig. 1).

**Table 3:** Frequency of identifying parotid lesions as Warthin tumors and malignant tumors depending on the cut-off values for T_peak_ and WR used as diagnostic criteria.

Cut-off value	Malignant tumors	Warthin tumors	*p* value
**T**_**peak**_** = 60 s**			
≤ 60 s	3/19	31/36	< 0.001
> 60 s	16/19	5/36	
**T**_**peak**_** = 120 s**			
≤ 120 s	8/19	34/36	< 0.001
> 120 s	11/19	2/36	
**WR = 30%**			
≤ 30%	18/19	16/36	< 0.001
> 30%	1/19	20/36	
**WR = 40%**			
≤ 40%	19/19	32/36	0.286
> 40%	0/19	4/36	
**WR = 13%**			
≤ 13%	16/19	5/36	< 0.001
> 13%	3/19	31/36

Subsequently, the accuracy of WR = 30% and WR = 40% for differentiating between malignant lesions and WTs was also analyzed. These cut-off values were previously used by Yabuuchi et al.^23^ (WR = 30%) and Hisatomi et al.^14^ (WR = 40%). WR = 30% was found to be a significant predictor of tumor type, enabling us to correctly identify 18/19 malignant lesions and 20/36 WTs (*p* < 0.001). ROC analysis revealed that DCE-MRI with this cut-off value provided 94.7% sensitivity and 55.6% specificity for identification of malignant tumors (AUC = 0.751, 95% CI 0.623–0.880) (Fig. 1). While cut-off of WR = 40% did not assist in discriminating between WTs and malignant lesions (*p* = 0.286) (Table 3).

During the next stage, the accuracy of TICs defined on the basis of the abovementioned cut-off values for T_peak_ and WR were analyzed. We defined TICs based on two sets of cut-off values: T_peak_ = 120 s/WR = 30% as previously used by Yabuuchi et al.^23^, and our original set T_peak_ = 60 s/WR = 30%. We did not analyze the sets with cut-off value WR = 40% since it did not significantly discriminate between WTs and malignant lesions. The distributions of TICs obtained on the basis of these two sets are presented in Table 3. The AUC for DCE-MRI with these cut-off values were 0.873 (95% CI 0.771–0.975) for T_peak_ = 60 s/WR = 30% and 0.854 (95% CI 0.748–0.960) for T_peak_ = 120 s/WR = 30% (Fig. 1). The sensitivity and specificity were 84.2% and 86.1% for T_peak_ = 60 s/WR = 30%, and 57.9% and 94.4% for T_peak_ = 120 s/WR = 30%, respectively.

Lastly, ROC analysis was conducted to identify cut-off values for T_peak_ and WR providing maximum sensitivity and specificity for identifying malignant tumors in our series. While the cut-off value for T_peak_ was the same as in previous studies dealing with the issue at hand (T_peak_ = 60 s), the cut-off value for WR was markedly lower (WR = 13%). Using this cut-off value, we were able to identify correctly 16/19 malignant lesions and 31/36 WTs (*p* < 0.001) (Table 3). ROC analysis showed that DCE-MRI with this cut-off value provided 84.2% sensitivity and 86.1% specificity in identification of parotid malignancies in our series (AUC = 0.852, 95% CI 0.736–0.968) (Fig. 1).

During the last stage, we verified the accuracy of TICs defined with the cut-off values T_peak_ = 60 s and WR = 13% (Table 4). The AUC with these cut-off values was 0.880 (95% CI 0.776–0.984) (Fig. 1), and the sensitivity and specificity were 78.9% and 88.9%, respectively.

**Table 4:** Frequency of identifying parotid lesions as Warthin tumors and malignant tumors depending on the type of TIC defined on the basis of the cut-off values for T_peak_ and WR.

Cut-off value	Malignant tumors	Warthin tumors	*p* value
**T**_**peak**_** = 60 s/WR = 30%**			
≤ 60 s/≤ 30%	2/19	11/36	< 0.001
> 60 s/> 30%	0/19	0/36	
≤ 60 s/> 30%	1/19	20/36	
> 60 s/≤ 30%	16/19	5/36	
**T**_**peak**_** = 120 s/WR = 30%**			
≤ 12 s/30%	7/19	14/36	< 0.001
> 120 s/> 30%	0/19	0/36	
≤ 120 s/> 30%	1/19	20/36	
> 120 s/≤ 30%	11/19	2/36	
**T**_**peak**_** = 60 s/WR = 13%**			
≤ 60 s/≤ 13%	1/19	1/36	< 0.001
> 60 s/> 13%	1/19	1/36	
≤ 60 s/> 13%	2/19	30/36	
> 60 s/≤ 13%	15/19	4/36	

## Discussion

Majority of the studies on the application of DCE-MRI in the differential diagnostics of parotid tumors are based on two parameters, T_peak_ and WR. As mentioned previously, these two parameters are sufficient for determining the type of TIC for a given lesion. Elmokadem et al. evaluated the value of multiparametric MRI for parotid tumor diagnostics. The study proved that the TIC type based on DCE-MRI significantly differentiates between benign and malignant lesions (*p* < 0.001) and has diagnostic accuracy of 96.55%. Additionally, no statistically significant difference was found between the ADC values of benign and malignant lesions^25^. Through our study, we aimed at determining whether the subset of “low” T_peak_ values traditionally assigned to both WTs and parotid malignancies^17,19–22^, includes some values which are specific solely for one of these groups. Our analysis demonstrates that the cut-off value for T_peak_ which most accurately differentiates between WTs and parotid malignancies was T_peak_ = 60 s. Using T_peak_ > 60 s as the only diagnostic criterion, we correctly identified 16/19 (84.2%) malignant tumors, while achieving false-positive results in 5/21 (23.8%). To the best of our knowledge, the role of T_peak_ as the only parameter of DCE-MRI for distinguishing between these tumors was studied in only a few studies^25–27^. Moreover, only Takashima et al. analyzed the accuracy of T_peak_ cut-off value at 60 s^24^. Using this cut-off value, they correctly identified 6/11 (54.5%) malignant parotid tumors, with the false-positive rate of 2/8 (25.0%).

**Figure 2:**
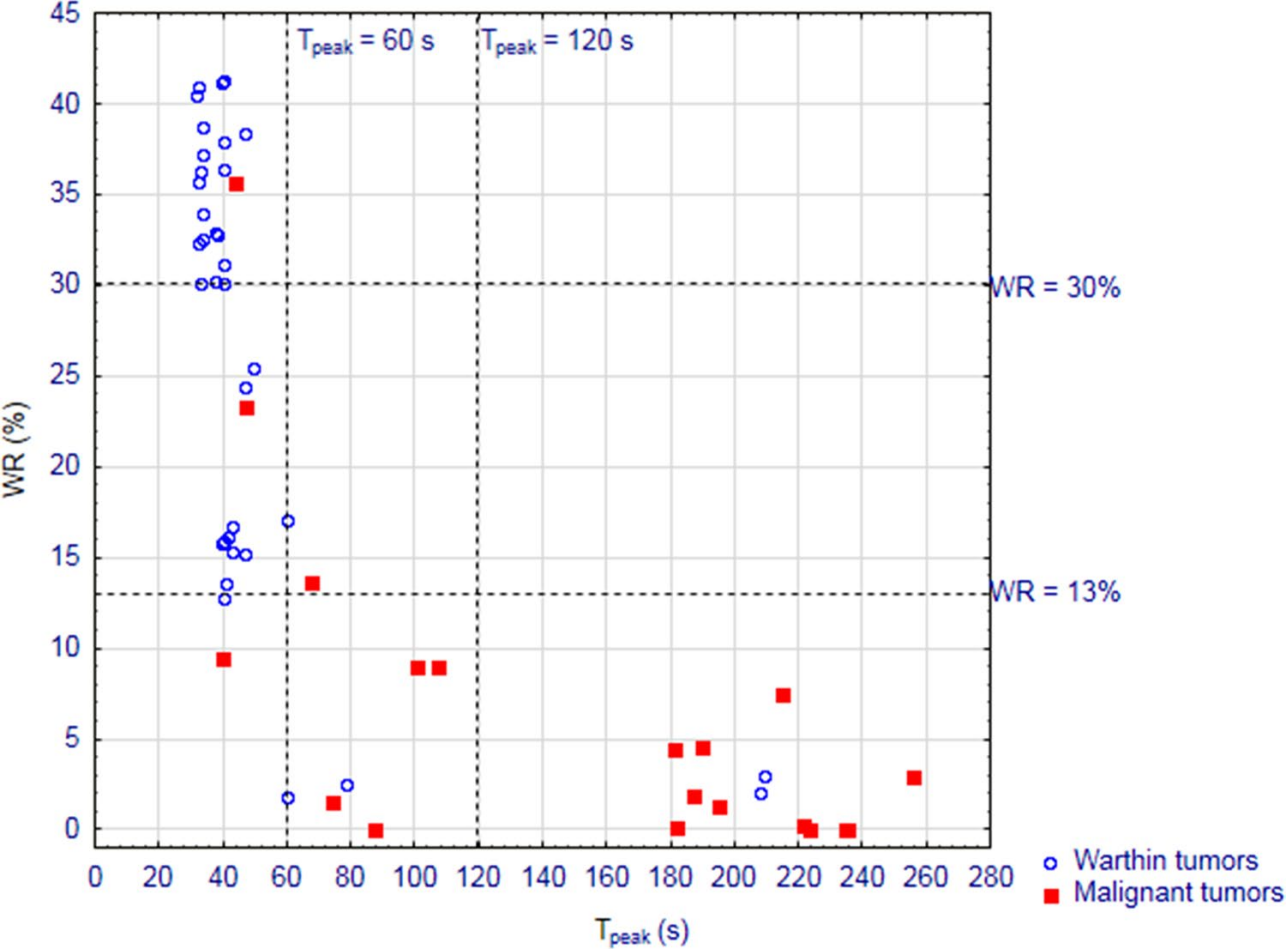
Distribution of analyzed Warthin tumors and parotid malignancies depending on their T_peak_ and WR values documented on DCE-MRI.

However, differential diagnostics of parotid tumors is typically based on simultaneous analysis of two DCE-MRI parameters T_peak_ and WR^28^. Therefore, our study additionally aimed at identifying a cut-off value for WR, which if analyzed in combination with T_peak_ = 60 s, would provide the highest accuracy for differentiating WTs and parotid malignancies based on radiological imaging. In previous studies on this topic, a lesion was considered malignant whenever its T_peak_ was > 120 s and its WR > 30% or > 40%. The principal limitation of these studies stemmed from the fact that they included PAs in addition to WTs and malignant lesions. Due to their specific microscopic structure, the TICs of PAs have markedly different characteristics. Our study, limited solely to WTs and malignant lesions, showed that the sensitivity of DCE-MRI for radiologically differentiating these lesions can be improved by 26% by using cut-off values T_peak_ = 60 s and WR = 30%. Importantly, ROC analysis revealed that the cut-off value for WR, which most accurately distinguishes WTs from malignant lesions was 13%. However, further analysis showed that an increase in AUC resulted due to a slight improvement in the specificity of DCE-MRI, but at the expense of its sensitivity (Fig. 2). Consequently, we did not find sufficient evidence to decrease the cut-off value for WR below 30%, especially since false-negative diagnosis of a malignant lesion as a WTs has considerably more devastating consequences than too extensive surgery inadvertently performed in the patient with false-positive diagnosis of a malignancy.

Besides showcasing a potential way to improve DCE-MRI accuracy for parotid tumor diagnostics, our study also revealed some drawbacks of this modality. To this date, majority of lesions with T_peak_ > 120 s were considered PAs^14,23^. However, our study showed that this cut-off value was also exceeded in as many as 11/19 (57.9%) parotid malignancy cases. This observation is consistent with the results of a small study conducted by Tsushima et al.^29^. Among nine parotid malignancies examined by DCE-MRI, the authors identified two adenoid cystic carcinomas whose TICs closely resembled those traditionally assigned to PAs (T_peak_ > 240 s). In our subset of 19 parotid malignancies, there were also two adenoid cystic carcinomas, one of which had T_peak_ value of 235 s. Altogether, these findings imply that characteristics of parotid tumors determined on DCE-MRI are likely modulated by their histological type and perhaps also clinical stage. Previous studies have showed that T_peak_ is inversely correlated with microvessel density in the examined tissue, and that WR increases proportionally to the connective tissue content^30^. Patella et al. investigated heterogeneity of intravoxel incoherent motion (IVIM) and DCE-MRI biomarkers in differentiating WTs and PAs. They found statistically significant differences for all histogram parameters and suggested that this was a result of wider capillary network in WTs than in PAs^31^. Based on this data, it can be hypothesized that some rapidly growing malignant tumors can present with extremely high T_peak_ and low WR, and as such may be misdiagnosed as PAs. Since only 1/19 parotid malignancy case in our series showed WR > 30%, this cut-off value seems to be a key diagnostic criterion for benign lesions. In the case of remaining tumors, the final decision on the type and extent of their resection should be established on the basis of cytological examination.

## Conclusions

The use of a different diagnostic algorithm than in the case of PAs, i.e., lower cut-off value for T_peak_ (= 60 s), appears to markedly increase the sensitivity of DCE-MRI in differentiating parotid malignancies and WTs. However, it is the WR value of > 30% which seems to be a key diagnostic criterion for benign lesions, as some parotid malignancies subjected to dynamic MRI may show features traditionally associated with PAs (T_peak_ > 120 s).
